# Evaluating hormonal mechanisms of vitamin D receptor agonist therapy in diabetic kidney disease: the VALIDATE-D study

**DOI:** 10.1186/1472-6823-13-33

**Published:** 2013-08-23

**Authors:** Jenifer M Brown, Kristina Secinaro, Jonathan S Williams, Anand Vaidya

**Affiliations:** 1 Division of Endocrinology, Diabetes, and Hypertension, Brigham and Women’s Hospital and Harvard Medical School, 221 Longwood Ave, RFB, Boston, MA 02115, USA

**Keywords:** Vitamin D, Calcitriol, Renin, Angiotensin, Diabetes, Kidney

## Abstract

**Background:**

Insufficient vitamin D status and increased renin-angiotensin system (RAS) activity have been associated with renal-vascular disease and nephropathy in diabetes. Accumulating evidence indicates that vitamin D receptor (VDR) activation lowers unfavorable RAS activity; however, more human intervention studies evaluating whether this mechanism could influence diabetic kidney disease are needed. We previously reported that both vitamin D levels and genetic variation at the VDR predict human RAS activity, and that vitamin D therapy can lower RAS activity in non-diabetics. The VALIDATE-D study is a randomized, placebo-controlled, intervention study designed to extend these findings by evaluating whether direct VDR activation in diabetes lowers circulating and local renal-vascular tissue RAS activity (Aims 1 and 2) in a manner similar to the action of ACE inhibitors (Aim 3).

**Methods/Design:**

Forty subjects with type 2 diabetes, microalbuminuria, and without chronic kidney disease will be recruited to undergo detailed assessment of the RAS before and after randomization to calcitriol 0.75 mcg/day or placebo. Primary analyses will evaluate whether calcitriol therapy reduces circulating and renal-vascular tissue-RAS activity in comparison to placebo. All subjects will thereafter be treated with lisinopril and followed for 3.5 months to evaluate whether combination therapy (calcitriol + lisinopril vs. placebo + lisinopril) additively or synergistically improves renal-vascular function, and lowers proteinuria.

**Discussion:**

The VALIDATE-D study is the first human intervention study to evaluate whether direct VDR activation can lower the human RAS in diabetes, compared to the effect of an ACE inhibitor, and whether this mechanism can translate to clinically relevant endpoints for diabetic kidney disease. The outcomes of VALIDATE-D will have major implications for the recommendation of vitamin D supplementation for the primary prevention of kidney complications in diabetes.

**Trial registration:**

ClinicalTrials.gov, NCT01635062

## Background

**Figure 1:**
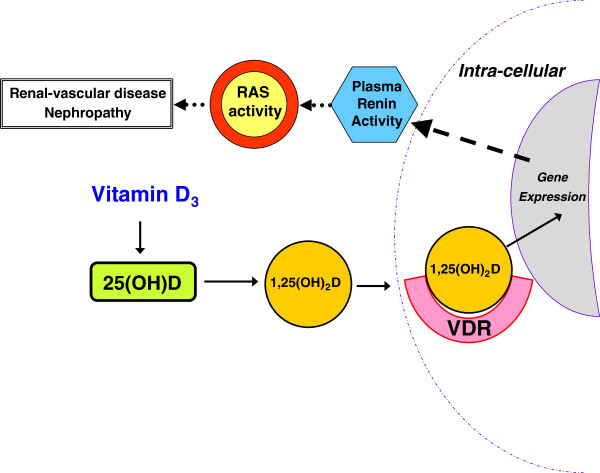
**The proposed interaction between vitamin D and RAS metabolism.** Renin catalyzes the conversion of angiotensinogen to angiotensin I, which is further converted to the vasoactive peptide angiotensin II. Angiotensin II is a direct vasoconstricter, and can also ilicit aldosterone secretion from the adrenal cortex. Under physiologic situations, activation of the RAS in response to renal-vascular hypo-perfusion serves to increase blood pressure and renal salt retention. However, in pathologic states (such as in diabetes and obesity), inappropriately high RAS activity contributes to vascular and kidney diseases. Vitamin D_3_ is largely produced in the skin with exposure to ultraviolet radiation, but may also be ingested orally. This precursor is hydroxylated to 25-hydroxyvitamin D (25[OH]D) and 25(OH)D serves as the stable barometer of clinical “vitamin D status.” Under the control of parathyroid hormone and calcium status, 25(OH)D can be hydroxylated to form the active vitamin D receptor (VDR) agonist 1,25-dihydroxyvitamin D (1,25[OH]_2_D). Activation of the VDR by 1,25(OH)_2_D is known to influence the regulation and expression of a myriad of genes, including renin.

The link between vitamin D and kidney disease, particularly in the setting of human diabetes, has received much attention [1,2]. Observational and limited interventional studies have supported a renoprotective benefit to higher vitamin D levels and vitamin D supplementation [3-7]; however, there is a need for more dedicated human intervention studies to confirm these findings. One of the main proposed mechanisms linking vitamin D with renal-vascular and kidney disease is regulation of the renin-angiotensin system (RAS) [8,9]. Since both of these hormonal systems have complex genetic and environmental regulatory steps (Figure 1), carefully controlled interventional studies that evaluate both *mechanisms of action and clinical outcomes* are needed to distinguish the hormonal interplay that is likely involved for vitamin D therapy to influence kidney disease.

Both excess activity of the renin-angiotensin system (RAS) and insufficient vitamin D status have been implicated in the development of renal-vascular disease that results in diabetic nephropathy [1,2,10]. The RAS can mediate renal-vascular disease via its circulating components, and additionally the locally expressed renal-vascular tissue-RAS also contributes to the development of kidney disease [11,12]. Excess renal-vascular tissue-RAS activity has been implicated in the development of diabetic nephropathy; RAS inhibitors [13-15], as well as favorable vitamin D status [5,16,17], may mitigate this effect.

Animal studies have shown that the activation of the vitamin D receptor (VDR) by 1,25-dihydroxyvitamin D (1,25[OH]_2_D) negatively regulates renin expression and thereby lowers RAS activity [9,18] (Figure 1). In mouse models of diabetes, both VDR-agonists and RAS-inhibitors blunted the development of diabetic nephropathy when given alone, but the combination (VDR-agonist + RAS-inhibitor) *synergistically* prevented the development of diabetic nephropathy via down-regulation of the renal-vascular tissue-RAS [19-21]. We, and others, have reported findings that translate these animal experiments to humans: the combination of vitamin D levels and genetic variation at the human VDR predicts RAS activity [22-24]. Large observational studies have shown that the prevalence of chronic kidney disease and proteinuria (a marker of kidney disease progression) are associated with lower 25(OH)D levels [3,25,26]. Due to their cross-sectional design, these aforementioned studies could not reveal causality or confirm the mechanism linking vitamin D-VDR interactions and renal outcomes. A few human interventions have demonstrated that VDR agonists may reduce proteinuria [2,7], but these studies were confined to populations with established chronic kidney disease (CKD), and evaluation of the RAS was not undertaken. We recently completed a pilot human intervention study in non-diabetics without CKD that demonstrated that high-dose vitamin D_3_ therapy improved renal-vascular hemodynamics by lowering renal-vascular tissue-RAS activity [5]. This latter effect was similar to that induced by an angiotensin converting enzyme (ACE) inhibitor, thereby further supporting a renoprotective effect of vitamin D therapy [5].

In light of these novel human results translated from animal experiments, we hypothesize that activation of the VDR represents a method to lower renal-vascular tissue-RAS activity in human diabetes, and could therefore play an important role in the *primary prevention* of diabetic nephropathy. We therefore designed the VALIDATE-D study: an ongoing randomized, double-blinded, placebo-controlled study to assess whether direct VDR activation: Aim 1) lowers circulating RAS activity in human diabetes; Aim 2) lowers renal-vascular tissue-RAS activity in human diabetes; and Aim 3) in combination with ACE inhibition, exerts an additive or synergistic effect on the renal-vascular tissue-RAS and proteinuria.

## Methods/Design

### Study population and recruitment

The VALIDATE-D study will enroll 40 adults, aged 18–70 years, with Type 2 diabetes (T2DM), microalbuminuria, and without known CKD (estimated glomerular filtration rate > 60 mL/min). Subjects will be recruited from the local community and from the outpatient clinics of the Division of Endocrinology, Diabetes, and Hypertension within the Department of Medicine at Brigham and Women’s Hospital, Boston, USA. Eligibility will be assessed at an in-person screening visit involving detailed history, physical examination, and laboratory assessment.

Inclusion criteria include T2DM that is controlled with diet, oral hypoglycemic agents, incretin analogues, or a single basal insulin injection; microalbuminuria; normal blood pressure or stage 1 hypertension treated with 0–1 antihypertensive agents; and normal blood cell counts, liver function, and electrocardiogram.

Exclusion criteria include CKD (glomerular filtration rate < 60 mL/min); poorly controlled T2DM (defined as HbA1c > 8.5% or the use of >1 daily insulin injection or a history or retinopathy); stage 2–3 hypertension or the use of more than one antihypertensive agent; kidney stones, parathyroid or granulomatous disorders, liver or heart failure, or coronary artery disease or congestive heart failure or cerebrovascular accidents. All subjects provide informed consent, and all study procedures have been approved by the Brigham & Women’s Hospital institutional and ethics board as well as the Brigham and Women’s Hospital Center for Clinical Investigation. Recruitment was initiated in September 2012 and is expected to be completed by September 2016.

### Study protocol overview

**Figure 2:**
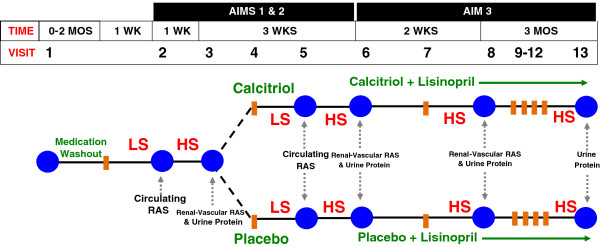
***VALIDATE-D study schema*: subjects will be screened and then may undergo a medication washout to withdraw interfering anti-hypertensive medications (when applicable).** Aim 1: The *circulating* RAS will be assessed on LS diet before and after randomization to calcitriol/placebo (visits 2 & 5). Aim 2: The *renal-vascular tissue-RAS* will be assessed on HS diet before and after calcitriol/placebo (visits 3 & 6). Aim 3: All subjects will then receive lisinopril 5 mg/d in addition to calcitriol/placebo to assess the impact of combination therapy (calcitriol + lisinopril vs. placebo + lisinopril) on the renal-vascular tissue-RAS (visits 6 & 8) and proteinuria (visits 6, 8, 13). Serum calcium and phosphate will be monitored weekly throughout the study for safety, at each visit and at designated safety checks (visits 4, 7, 9–12).

A detailed study schema is shown in Figure 2. All study visits will occur in the Clinical Research Center (CRC) at Brigham and Women’s Hospital. Studies will be performed in the CRC in order to control posture, time of day, dietary intake, and medication administration, since all of these factors are known to influence RAS activity. In brief, detailed profiling of the RAS will be performed at baseline before any intervention (Visits 2 & 3), again after double-blinded randomization to calcitriol or placebo (Visits 5 & 6) to assess whether calcitriol lowers RAS activity, and again after the addition of lisinopril to the blinded study drug to assess for an additive or synergistic relationship between VDR-agonist and ACE inhibitor therapy on the RAS and proteinuria (Visits 8 & 13).

#### Washout

It is well known that many medications interfere with physiologic RAS responses or with the assays that measure the RAS. To optimize the reliability and accuracy of RAS measurements, medications that interfere with RAS assessments will be withdrawn. Subjects using RAS inhibitors (such as angiotensin converting enzyme [ACE] inhibitors, angiotensin receptor blockers, or mineralocorticoid receptor antagonists) will stop their medications for 2 months prior to study. Subjects using beta-blockers or diuretics will stop their medications for 1 month prior to study, whereas those on calcium channel blockers will stop for 2 weeks prior to study. Medication washout will be supervised by a study staff based on previously described safety protocols [5] after providing and instructing each subject on how to use a home sphygmomanometer. As previously described, in cases where hypertension (159/99 mmHg) results following withdrawal of an antihypertensive medication, amlodipine will be used to lower blood pressure as it has a neutral effects on RAS activity.

#### Study diets

All study procedures will be conducted using one of two specifically designed study diets.

### Low-salt diet

The low salt (LS) diet includes 10 mEq of sodium, 100 mEq of potassium, and <600 mg of calcium per day. The purpose of this diet is to induce *maximal stimulation* of RAS components to allow for adequate measurements of the *circulating RAS*. Subjects will consume the LS diet for 1 week prior to study visits where the circulating RAS will be measured (Visits 2 and 5). All daily meals and food intake during the LS dietary phase will be prepared and provided to study participants by the CRC dietary kitchen staff.

### High-salt diet

The high sat (HS) diet includes 200 mEq of sodium, 50 mEq of potassium, and <600 mg of calcium per day. The purpose of this diet is to induce *maximal suppression* of the circulating RAS components in order to most effectively measure the *renal-vascular tissue-RAS*, as previously described [5,17]. Subjects will consume the HS diet for 1 week prior to study visits where the renal-vascular tissue-RAS will be evaluated (Visits 3, 6, and 8). The HS diet involves consumption of an ad lib diet, with supplements provided to achieve the sodium and potassium contents.

### Low calcium diet

Subjects will be instructed and taught to restrict dietary calcium intake during the LS and HS dietary phases (<600 mg/day), and also during the duration of the study in between study visits (<1000 mg/day). The purpose of calcium restriction is to minimize the risk of hypercalcemia and hyperphosphatemia associated with calcitriol administration; this risk is negligible in the context of low dietary calcium intake [27-29]. A registered dietician will coach subjects throughout the study on restricting calcium intake and will provide materials to guide dietary intake at home (Dietary Calcium Intake Recommendations Additional file 1). Compliance with study diets will be confirmed by 24-hour urine collections obtained at every study visit.

#### Baseline assessments of the circulating and renal-vascular tissue-RAS (Visits 2 & 3)

After a screening visit (Visit 1) and a potential antihypertensive washout phase, all subjects will undergo baseline assessments to characterize the circulating and renal-vascular tissue-RAS.

### Circulating RAS on LS diet (Visit 2)

Study visit 2 is designed to measure the baseline circulating RAS under conditions designed to induce maximal stimulation of the circulating RAS: restricted dietary sodium intake and upright posture. After one week of LS diet consumption, subjects are studied at 8 AM in the morning (Figure 2). Subjects will maintain an upright standing posture for at least 1 hour. Blood samples are obtained at the end of the postural phase to measure circulating RAS components (plasma renin activity, angiotensn II, aldosterone).

### Renal-vascular tissue-RAS on HS diet (Visit 3)

**Figure 3:**
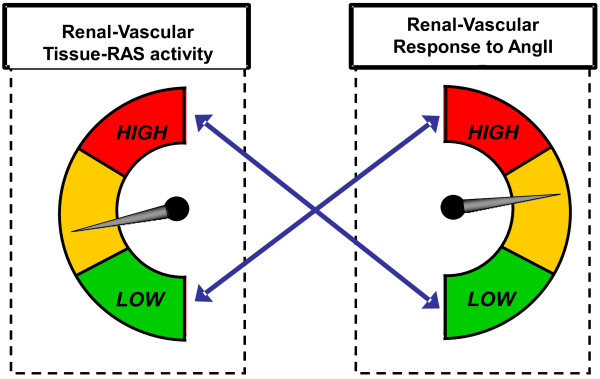
**Reciprocal relationship between renal-vascular tissue-RAS activity and the renal-vascular response to angiotensin II.** A blunted response to angiotensin II reflects a high level of local, endogenous, renal-vascular tissue-RAS activity (the smaller the decline in renal plasma flow in response to an infusion of angiotensin II, the greater the activity of the local renal-vascular tissue-RAS). Conversely, a robust response to angiotensin II is observed when tissue-RAS activity is low.

Study visit 3 is designed to assess baseline renal-vascular tissue-RAS activity by measuring changes in renal plasma flow (RPF) in response to an infusion of exogenous angiotensin II. Angiotensin II is a vasopressor that specifically interacts with angiotensin receptors to induce renal-vascular vasoconstriction resulting in a decrease in RPF. *The magnitude of the decline in RPF to angiotensin II is inversely related to renal-vascular tissue-RAS activity* (Figure 3); greater renal-vascular responses to angiotensin II signify lower local tissue-RAS activity [5,30,31]. Using this physiologic methodology, measurement of RPF responses to angiotensin II provide an indirect method to assess the human renal-vascular tissue-RAS.

To adequately measure the renal-vascular tissue-RAS, subjects are studied on a HS diet and in overnight supine posture to suppress endogenous circulating RAS components, thereby amplifying measurable RPF responses to angiotensin II [32]. After completing one week of HS diet, subjects will be admitted to the CRC and maintained supine overnight and throughout the study procedures on the following morning. In the morning, two intravenous lines will be placed to facilitate infusion of study drugs and frequent blood sampling. Weight-based *para-*aminohippurate (PAH) will then be infused and its clearance used to measure RPF throughout the duration of the study visit [5]. After allowing PAH concentrations to reach a steady state over 60 minutes, blood will be sampled to establish the baseline RPF. Angiotensin II will then be infused at 0.3 ng/kg/min for 45 minutes and escalated to 1 ng/kg/min for an additional 45 minutes to assess dose–response characteristics. PAH will be measured during and after each angiotensin II infusion to measure RPF dynamics. Blood pressure will be monitored closely throughout the infusions based on standardized safety protocols [5] that we have extensive experience performing in our CRC. The study visit will end upon completion of the angiotensin II infusions, and subjects will be discharged to undergo randomization to the study drug.

#### Randomization to study drug

Following study visit 3, subjects will be randomized to either calcitriol 0.75 mcg/day or placebo arm using a 2 block randomization. Double-blinded randomization will be performed by the investigational drug pharmacy at our institution, and neither the subjects nor the investigators will know the identity of the randomized study drug.

#### The influence of calcitriol on the RAS (Visits 5 & 6)

### Circulating RAS on LS diet (Visit 5)

After 2 weeks of daily calcitriol or placebo, subjects will return on LS diet and complete visit 5, which is identical to visit 2. Aim 1 of this study (to assess whether direct VDR activation lowers circulating RAS activity in human diabetes) is executed in study visits 2 and 5. *Anticipated Results:* It is anticipated that calcitriol therapy will lower circulating RAS components (plasma renin activity and angiotensin II) when compared to placebo, demonstrating for the first time that VDR agonism can inhibit the circulating RAS in human diabetes.

### Renal-vascular tissue-RAS on HS diet (Visit 6)

After a third week on the study drug, subjects will return on HS diet for an overnight CRC study visit 6, that is identical to Visit 3. This visit will permit assessment of study Aim 2 (to assess whether direct VDR activation lowers renal-vascular tissue-RAS activity in human diabetes) by comparing the renal-vascular tissue-RAS activity before and after calcitriol/placebo. *Anticipated Results:* It is anticipated that calcitriol therapy will raise basal RPF and increase the RPF sensitivity to angiotensin II, thereby demonstrating that VDR activation improves renal-vascular hemodynamics by lowering local renal-vascular RAS activity in human diabetics without CKD.

#### The influence of combination VDR-agonist and ACE inhibitor therapy on the renal-vascular tissue RAS (Visit 8)

Upon completing study visit 6, all subjects will receive lisinopril (initially 2.5 mg daily, which will be increased to 5 mg daily after one week, unless blood pressures are below 90/50 mmHg or subjects experience orthostatic symptoms) in addition to their randomized study drug (Figure 2). After 2 weeks of daily combined therapy (calictirol + lisinopril or placebo + lisinopril), subjects will return on the HS diet for a final inpatient CRC visit that is identical to study visits 3 & 6. Comparing the outcomes of study visits 6 and 8 will permit evaluation of Aim 3 of this study (to assess whether direct VDR activation in combination with ACE inhibition exerts an additive or synergistic effect on the renal-vascular tissue-RAS and proteinuria). *Anticipated Results:* Combination therapy (calcitriol + lisinopril) will additively or synergistically raise RPF and increase the RPF sensitivity to angioensin II infusion, reflecting decreased renal-vascular tissue-RAS activity when compared to placebo + lisinopril.

#### The influence of chronic combination therapy on proteinuria (Visit 13)

Following completion of study visit 8, subjects will be discharged home to continue both the study drug and lisinopril for three months of long-term therapy (Figure 2). For the final visit of the study, subjects will return after one week of HS diet with a completed 24-hour urine collection, which will be analyzed for protein. The 24-hour urine protein measured at this visit will be compared using repeated measures analyses to the urine protein levels obtained from 24-hour urine collections completed at each inpatient visit on HS dietary balance (visits 3, 6, 8). *Anticipated Results:* Combination therapy (calcitriol + lisinopril) will additively or synergistically lower proteinuria when compared to placebo + lisinopril, thereby paralleling prior animal studies that showed that the combination of VDR agonist + conventional RAS inhibitor therapy induced synergistic renoprotective benefits in mouse models of diabetes [19-21].

### Safety & monitoring

#### Calcium/Phosphate

To minimize the risk of hypercalcemia due to calcitriol, all subjects will undergo laboratory evaluation of serum calcium and phosphate at 1–3 week intervals throughout the study duration, including at the aforementioned study visits and additionally at designated safety visits (visits 4, 7, 9–12). If calcium or phosphate rises above the upper limit of normal, the test will be repeated in 3 days and subjects will be counseled by our dedicated study dietician on adherence to a low calcium diet (Dietary Calcium Intake Recommendations Additional file 1). If calcium or phosphate levels remain elevated, or the calcium-phosphate product exceeds 50, the dose of study drug will be reduced by one third. Based on several prior studies that have employed chronic VDR-agonist therapy in combination with lowered dietary calcium intake, our dosing and study duration are unlikely to invoke hypercalcemia or hyperphosphatemia [27-29].

#### Blood pressure

During PAH and angiotensin II infusion, blood pressure (BP) will be monitored every 2 minutes using an automated sphyngomanometer. An increase in BP is an expected effect of angiotensin II infusion, and the following protocol has been used safely in over 1500 subjects [5]. The 0.3 ng/kg/min dose was selected because it does not induce a pressor effect but still influences renal-vascular hemodynamics [30], and the 1 ng/kg/min dose was selected because it has previously been shown to induce minimal BP elevations well within a safety range [5,30]. A study physician will supervise all angiotensin II infusions as previously described [5].

All subjects will monitor their BP daily with a home sphygmomanometer while on the washout phase (when applicable) and while on lisinopril and will communicate their readings to study staff twice weekly. Study staff will monitor for unsafe high BP during the washout period (>159/99 mmHg) or unsafe hypotension during lisinopril treatment (<90/50 mmHg). Subjects who develop high BPs during the washout period may be treated with amlodipine which has a neutral effect on RAS components. Subjects who develop low BPs on lisinopril may have their dose reduced 50%, however, if a 2.5 mg daily dose is not tolerated, subjects will be removed from the study.

#### Blood glucose

Subjects will be asked to check their home blood glucose daily, and study staff will monitor their glycemic control throughout the study. If fasting morning blood glucose is consistently >150 mg/dL, medications will be adjusted by a study physician. Such adjustments include increasing the dose of a home medication or adding a second oral hypoglycemic agent.

#### Lipids

Any subject who has a screening LDL cholesterol greater than 100 mg/dL, will either be prescribed simvastatin 20-40 mg daily, or if already taking a statin medication, have their pre-existing statin dose increased, to remain consistent with clinical standards of care for diabetes.

### Laboratory measurements

25(OH)D (Diasorin, Inc., Stillwater, MN), 1,25(OH)_2_D (Diasorin), parathyroid hormone (Beckman Coulter, Fullerton, CA), and serum and urine electrolytes (including calcium and phosphate) will be measured at baseline at all visits. Components of the circulating RAS, including plasma renin activity (Diasorin) and aldosterone (Siemens, Los Angeles, CA), will be measured from samples drawn in upright and supine posture (Visits 2, 5, 13) and before and during angiotensin II infusion (Visits 3, 6, 8) [5]. For assessment of RPF, *para*-aminohippurate will be measured in triplicate at steady-state and during angiotensin II infusion and will then be adjusted by body surface area in all RPF calculations (Visits 3, 6, 8).

### Statistical analyses

#### Sample size calculations

Sample size calculations were made using our prior data evaluating the impact of vitamin D on the systemic [23] and renal-vascular tissue-RAS [5] by paired analyses with an alpha-error of 0.05. For Aim 1, prior data suggest that therapy with a VDR agonist could reduce plasma renin activity, a measure of the circulating RAS, by 1 ng/mL/hr (SD = 0.54). A minimum of 5 subjects would be required in each study arm for a paired comparison to detect this difference with a power of 0.9. In our pilot intervention study, we demonstrated an effect of vitamin D therapy on the renal-vascular tissue-RAS (Aims 2 & 3) in a sample of 14 subjects. To account for underestimation of sample-size, variability in measurements, and to permit non-paired comparisons, we intend to study 20 subjects in each intervention group.

#### Outcomes

Paired and repeated measures analyses will be used to evaluate intra-individual changes in the circulating RAS, RPF responses to angiotensin II infusion (i.e. renal-vascular tissue-RAS), and urine protein levels. Non-paired comparisons will be used to evaluate changes in these parameters between the calcitriol and placebo groups. A two-tailed *P*-value < 0.05 will be considered statistically significant.

## Discussion

The VALIDATE-D study is a unique randomized and double-blinded human intervention study that is designed to elucidate the *mechanism* by which vitamin D therapy may impart renoprotective benefits in diabetes. Vitamin D metabolism and regulation of the RAS are complex processes that involve multiple genes, gene-products, and environmental inputs (Figure 1); the genetic variation, dietary intake, and other environmental exposures for each individual have been shown to alter the balance of each of these hormonal systems [33-35]. In this regard, using supplementation with vitamin D_3_ in an intervention study to induce a clinical outcome that is mediated by the interaction between 1,25(OH)_2_D and the VDR (such as RAS inhibition to influence renal-vascular function) is analogous to administering a pre- or pro-drug that is dependent on multiple subsequent enzymatic steps, each regulated and modified by genetics and environment. The strengths of the VALIDATE-D study are that it focuses on overcoming this individual variability by investigating the influence of *direct* VDR-agonist therapy in modulating RAS activity and renal-vascular function, and employs a meticulous design to evaluate the underlying mechanism of potential findings (regulation of RAS activity).

Increasing evidence highlights a relationship between vitamin D and the RAS, with relevant implications for renal-vascular and kidney disease [1,36]. Lowering unfavorable RAS activity has known vascular benefits in diabetes [37], and the use of RAS inhibitors is common practice to prevent or delay the progression of diabetic nephropathy. Animal studies have elegantly shown that activating the VDR results in lowering of the renal-vascular tissue-RAS, and that this interaction translates to reduced kidney injury, particularly in the setting of animal models of diabetes [19-21]. While human studies often focus on influencing clinical outcomes by supplementing with vitamin D_3_[5,38], research studies that employ direct VDR-agonists may be most effective at invoking VDR-mediated effects such as RAS modulation [2,39]. The advantage of using direct VDR-agonists in intervention studies is that they likely maximize any effect mediated by the VDR, eliminate inter-individual variations in vitamin D metabolism, and may influence clinical outcomes in a shorter duration of time than would take with vitamin D_3_ supplementation. The disadvantage of using VDR-agonists is that they are not commonly used in individuals without CKD and therefore direct clinical translation of study findings is limited; however, positive findings would support any therapy (vitamin D_3_ or VDR-agonist) that raises 1,25(OH)_2_D levels or activates the VDR as an equal surrogate.

The results of the VALIDATE-D study will shed light on: 1) the mechanism by which VDR activation influences renal-vascular function in human diabetes (via modulation of the RAS), and 2) whether VDR activation modulates renal hemodynamics and proteinuria in human diabetes. These findings would not only carry major implications for the recommendation of vitamin D supplementation in the primary prevention of diabetic nephropathy, but they could help interpret the findings of many other studies that evaluated clinical outcomes of vitamin D therapy without accounting for the mechanisms involved. In this regard, the VALIDATE-D study may explain the reasons prior negative studies failed to see outcomes and why positive studies may have succeeded.

At least three unique findings will emerge from the VALIDATE-D study. First, this intervention study will conclusively evaluate whether vitamin D therapy lowers the *circulating* human RAS. By using direct VDR agonists and a meticulous study design to account for confounders of the RAS (diet, posture, medications, time of day), this study will investigate whether calcitriol therapy lowers the maximally stimulated RAS on a LS diet in comparison to placebo. Second, this study will evaluate whether direct VDR activation improves renal-vascular hemodynamics by increasing renal blood flow and the renal-vascular sensitivity to angiotensin II; both of these observations are consistent with reductions in the renal-vascular tissue-RAS as would be induced by an ACE inhibitor. The study design includes a comparison of renal-vascular hemodynamics (while the circulating RAS is suppressed on a HS diet) during treatment with calcitriol vs. placebo, and then again during treatment with calcitriol + lisinopril vs. placebo + lisinopril (Figure 2). In this regard, the study design is optimized to decipher the individual role of VDR activation, and the effect of combining VDR activation with conventional RAS inhibition, since this combination therapy has been shown to provide synergistic renoprotection in animal models of diabetes [19-21]. The assessment of the renal-vascular tissue-RAS in humans is a unique and defining aspect of this study protocol. The renal vasculature is specifically sensitive to the effects of angiotensin II, and the magnitude of renal-vascular responses to angiotensin II is inversely proportional to the degree of local tissue-RAS activity [5,11,16]. High endogenous tissue-RAS activity results in a blunted local sensitivity to the effect of angiotensin II, while inhibiting the local renal-vascular tissue-RAS (for example with an ACE inhibitor) improves the renal-vascular sensitivity to angiotensin II [30] (Figure 3). Therefore, our ability to measure renal hemodynamics via *para*-aminohippurate clearance permits us to objectively ascertain the state of the renal-vascular tissue-RAS in response to drug intervention (calcitriol, lisinopril, placebo) and in response to provocation by angiotensin II. Lowering renal-vascular tissue-RAS activity in pathologic states such as diabetes and obesity with conventional pharmacotherapy (ACE inhibitor or angiotensin receptor blocker) has shown efficacy for the primary prevention of diabetic nephropathy [15,40,41]. Thirdly, the impact of adding chronic VDR-agonist therapy to ACE inhibitor therapy on proteinuria will be assessed. Since the study population is strategically selected to have diabetes without CKD, together these study findings will have implications for recommending vitamin D supplementation to lower unfavorable RAS activity for the *primary prevention* of diabetic nephropathy.

We have previously shown in a pilot study that high-dose vitamin D_3_ therapy to markedly raise 1,25(OH)_2_D concentrations can lower renal-vascular tissue-RAS activity in non-diabetics with normal renal function [5]. Like an ACE inhibitor, this intervention induced a favorable state of low tissue-RAS resulting in increased renal-vascular sensitivity to angiotensin II [5]. The VALIDATE-D study will extend these findings by employing a double-blinded randomization, employing direct VDR agonist therapy, and focusing on patients with diabetes without CKD. In prior clinical studies of secondary prevention in diabetes with pre-existing CKD, direct VDR agonists (calcitriol and paracalcitol) reduced proteinuria [7,42-45]; whether this effect was mediated by the RAS, or could be extrapolated for the *primary* prevention of diabetic nephropathy, are questions that the VALIDATE-D study will clarify.

VALIDATE-D is the first human study to investigate whether vitamin D therapy to activate the VDR can lower circulating and renal-vascular tissue-RAS activity in diabetics without CKD. This study is designed to specifically focus on whether modulating the RAS represents a key mechanism by which vitamin D intervention impacts renal-vascular hemodynamics and kidney function. In this regard, VALIDATE-D will provide evidence that may influence clinical recommendations for maintaining vitamin D status for the *primary prevention* of diabetic nephropathy. In addition, VALIDATE-D will examine whether the action of potent VDR agonist therapy complements the known beneficial effects of ACE inhibition, as has been observed in animal models [19]. If so, the results of VALIDATE-D could influence pharmacologic practices by favoring vitamin D_3_ supplementation instead of, or in addition to, ACE inhibitor therapy for patients with diabetes.

## Competing interests

The authors affirm they have no competing interests.

## Authors’ contributions

AV developed the study design and concept and is involved in the conduct and supervision of the study as the principal investigator. JMB and JSW contributed to the study design and study conduct. KS is involved in recruiting subjects and providing study coordination and dietary input. All authors contributed to the writing and editing of this manuscript.

## Authors’ information

The authors have unique experiences in conducting human physiology studies such as the one described here. AV and JSW have nearly ten years of experience designing and performing human research focused on the renin-angiotensin sytem and calcium-regulatory hormones. This includes research protocols with detailed dietary control and modulation of hormones using Clinical Research Center Resources. AV, JSW, and JMB have published on interactions between the renin-angiotensin system and calcium-regulatory hormones, and their reputation in this field has resulted in invitations to give regional and national talks on this topic.

## Pre-publication history

The pre-publication history for this paper can be accessed here:

http://www.biomedcentral.com/1472-6823/13/33/prepub

## Supplementary Material

Additional file 1 Dietary Calcium Intake Recommendations. Click here for file
