# Supplementary material for: Evaluating hormonal mechanisms of vitamin D receptor agonist therapy in diabetic kidney disease: the VALIDATE-D study
Source: BMC Endocr Disord. 2013 Aug 23;13:33. doi: 10.1186/1472-6823-13-33 (PMC3765219; doi:10.1186/1472-6823-13-33)
Supplement: Additional file 1 — Dietary Calcium Intake Recommendations. [file 1472-6823-13-33-S1.docx]

**Dietary Calcium Intake Rcommendations**

**150-200mg Calcium**

**Choose________per day from this box**

1 oz (slice) cheese

1 cup cottage cheese

3 oz Salmon

1 cup broccoli, cooked

1 cup arugula, raw

1/2 cup collards, cooked

1/4 block tofu

**50-100mg Calcium**

**Choose_______per day from this box**

1 cup chickpeas, garbanzo beans

½ cup white beans

1 medium orange

1 oz almonds

1 cup kale, raw

1 cup turnips, raw

1 egg

½ cup ice cream

4 oz ready to eat pudding

2 slices whole wheat bread

**>300mg Calcium**

**Choose_______per day from this box**

1 cup yogurt

1/2 cup ricotta cheese

1 cup calcium-fortified cereal (ex. TOTAL)

**250-300mg Calcium**

**Choose_______per day from this box**

1 cup of milk

1 cup spinach, frozen, cooked

1 cup calcium-fortified orange juice
